# Simultaneous Voltammetric Detection of Carbaryl and Paraquat Pesticides on Graphene-Modified Boron-Doped Diamond Electrode

**DOI:** 10.3390/s17092033

**Published:** 2017-09-06

**Authors:** Aniela Pop, Florica Manea, Adriana Flueras, Joop Schoonman

**Affiliations:** 1 Department of Applied Chemistry and Environmental Engineering and Inorganic Compounds, Politehnica University of Timisoara, P-ta Victoriei No. 2, 300006 Timisoara, Romania; aniela.pop@upt.ro (A.P.); adriana.balasoiu@ymail.com (A.F.); 2 Faculty of Applied Sciences, Department of Chemical Technology, Section Materials for Energy Conversion and Storage, Delft University of Technology, Lorentzweg 1, 2628 CJ Delft, The Netherlands; J.Schoonman@tudelft.nl

**Keywords:** direct electrochemical detection, carbaryl, paraquat, simultaneous detection, pesticide residues, natural juice

## Abstract

Monitoring of pesticide residues in food, beverages, and the environment requires fast, versatile, and sensitive analyzing methods. Direct electrochemical detection of pesticides could represent an efficient solution. Adequate electrode material, electrochemical technique, and optimal operation parameters define the detection method for practical application. In this study, cyclic voltammetric and differential pulse voltammetric techniques were used in order to individually and simultaneously detect two pesticides, i.e., carbaryl (CR) and paraquat (PQ), from an acetate buffer solution and also from natural apple juice. A graphene-modified boron-doped diamond electrode, denoted BDDGR, was obtained and successfully applied in the simultaneous detection of CR and PQ pesticides, using the differential pulse voltammetric technique with remarkable electroanalytical parameters in terms of sensitivity: 33.27 μA μM^−1^ cm^−2^ for CR and 31.83 μA μM^−1^ cm^−2^ for PQ. These outstanding results obtained in the acetate buffer supporting electrolyte allowed us to simultaneously detect the targeted pesticides in natural apple juice.

## 1. Introduction

Pesticide residues are a concern to human health, and the monitoring of these residues in foodstuffs and water represents one of the most important steps in minimizing potential hazards to consumers [1]. In order to avoid the health hazards caused by pesticide residues, governments and international organizations regulate the maximum residues level (MRL) in fruit and vegetables, aiming to limit population exposure and to confirm the proper use and exact concentrations of pesticides in agricultural products [2]. The MRL values established by regulatory bodies range between 0.01–10 mg/kg, in relation to matrices and acceptable daily intake [3,4]. Also, 10 μg L^−1^ for paraquat and 90 μg L^−1^ for carbaryl have been reported as guideline limits for water [5]. Consequently, trace level detection and fast identification represent important aspects in analytical method selection. In general, pesticides are detected in food and water using chromatographic analytical methods such as gas and liquid chromatography in tandem with mass spectrometry [1,3,4,6], immunoassays and immunosensors [7], flow injection chemiluminescence [8], or fluorescence [9]. The main disadvantages of these methods are the time consumption, including prerequisite sample pre-treatment/pre-concentration, the expensive instrumentation and the toxic organic reagents required. The electrochemical method has been extensively studied lately as a viable alternative [10]. In addition, the enzymatic sensor exhibits specificity towards a certain species; this specificity inhibits development of the methodology for the simultaneous detection of pesticides. The advantages of applying electrochemical methods in pesticide residues detection are the low cost, easy operation, fast response, and high sensitivity [6,11,12].

The key to successful application of electrochemical methods in pesticides detection is adequate electrode material. The boron-doped diamond electrode (BDD) is recognized as one of the most versatile electrode materials used in electroanalysis, due to its wide potential window in aqueous solution, low background currents, and long-term stability [13,14,15]. Very promising results have been found for BDD application in the electrochemical detection of pesticides [16,17,18,19,20,21]. Carbon nanomaterials such as graphene, fullerene, and carbon nanotubes are used to enhance the electroanalytical performances of the working electrodes [11,22]. The unique morphological characteristics, chemical stability, and electrochemical properties of graphene open the possibility of implementing the direct electron-transfer-based mediatorless electrochemical detection scheme [22,23]. The large surface area combined with the excellent electrochemical properties of graphene should enhance the transfer electron rate and, as a consequence, the detection sensitivity [22]. In this study, the graphene-modified boron-doped diamond electrode-based electrochemical detection method was developed to simultaneously detect carbaryl (CR) and paraquat (PQ) in aqueous solution. This method was validated by application to natural apple juice, without any pre-purification step. To the best of our knowledge, no methodology for the simultaneous electrochemical detection of carbaryl and paraquat has been reported.

Carbamate compounds are the most used insecticides in agriculture, and their presence in the environment could represent a serious threat to human health [10,19,24,25]; from this class, carbaryl (CR) has been chosen as a model in our study. Another target pesticide was paraquat (PQ), considered one of the most hazardous compounds to human health [14], with a widespread use and a long resistance time [26]. Cyclic and differential pulse voltammetry were applied to design the optimum procedure for the fast simultaneous detection of both carbaryl and paraquat in buffer solution. The simultaneous detection method was validated through its application to real apple juice.

## 2. Materials and Methods

All the electrochemical measurements were performed using an Autolab potentiostat/galvanostat PGSTAT 302 (Metrohm Autolab B.V., Utrecht, The Netherlands) controlled with GPES 4.9 software using a three-electrode cell, with a saturated calomel reference electrode, a platinum counter electrode and a graphene-modified boron-doped diamond (BDDGR) working electrode. The commercial BDD disc surface electrode with a boron content of about 0.1% and a diameter of 3 mm, provided by Windsor Scientific Ltd., (Slough Berkshire, UK), was modified using graphene oxide solution (4 mg mL^−1^) provided by Merck, through electrochemical deposition at a potential of −1.2 V/SCE for 60 s. The supporting electrolyte used in the study was acetate buffer, with a pH of 5.6. The acetate buffer was obtained by mixing 27.215 g sodium acetate, 6 mL of glacial acetic acid and 200 mL acetonitrile with distillated water for a total volume of 1000 mL solution. The potential range was established between −1.00 and +1.75 V/SCE and, prior to use, the working electrode was stabilized through 15 continuous repetitive cyclic voltammograms running, in order to obtain a stable and reproducible background current.

The carbaryl (CR) and paraquat (PQ) were PESTANAL analytical standards, 99.9% purity (Sigma Aldrich, Schnelldorf, Germany). A 1 mM CR solution was prepared using acetonitrile as the solvent, and a 1 mM PQ solution was prepared with distillated water.

The electrochemical techniques applied were cyclic voltammetry, with a scan rate of 50 mV s^−1^, and differential pulse voltammetry, with two different operating conditions: (1) step potential of 6 mV, modulation amplitude of 800 mV, modulation time of 0.004 s and (2) step potential of 5 mV, modulation amplitude of 50 mV, modulation time of 0.004 s.

The electroanalytical parameters—the limit of detection (*LOD*) and the limit of quantification (*LOQ*)—were calculated as follows:

*LOD* = 3 × *SD*/*m* (1)

*LOQ* = 10 × *SD*/*m* (2)

where *SD* is the standard deviation of the 6 blanks and *m* is the slope of the calibration plot [1,27].

## 3. Results and Discussion

To develop the protocol for the simultaneous detection of carbaryl and paraquat, the respective electrochemical behavior of carbaryl and paraquat was investigated on the graphene-modified boron-doped diamond electrode (BDDGR) in comparison with the commercial BDD electrode.

### 3.1. Individual Detection of Carbaryl and Paraquat

#### 3.1.1. Cyclic Voltammetry

**Figure 1:**
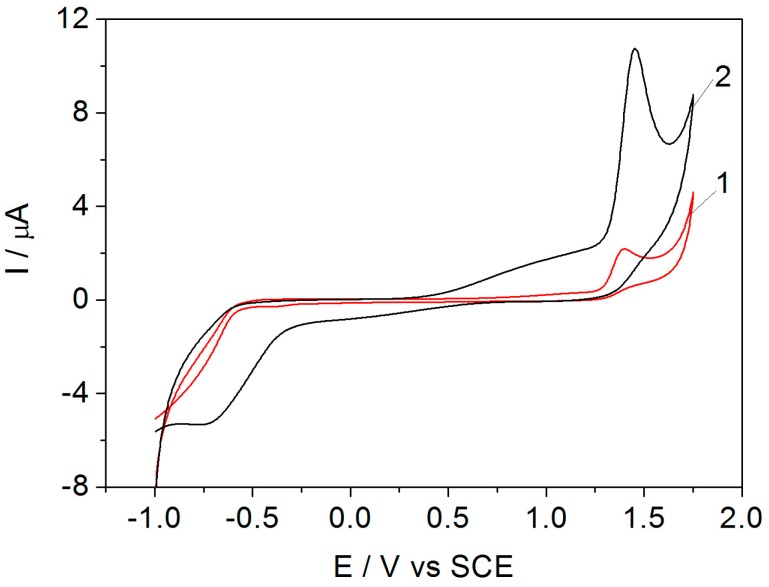
Cyclic voltammograms recorded on boron-doped diamond (BDD) (curve 1) and graphene-modified boron-doped diamond (BDDGR) (curve 2) electrodes in sodium acetate buffer supporting electrolyte and in the presence of 50 μM carbaryl (CR), scan rate 50 mV s^−1^.

Taking into account the promising results related to the electrochemical detection of carbaryl in natural waters on a boron-doped diamond electrode [28], the comparative electrochemical behavior of carbaryl on BDDGR and commercial BDD was investigated through recording voltammograms DD in the presence of 50 μM carbaryl to check the effect of graphene on carbaryl electrooxidation and, implicitly, its detection signal. A larger background current that corresponds to larger active surface area and an approximately 4 times higher useful signal is noticed for BDDGR (Figure 1); this shows the electrocatalytic effect of graphene on carbaryl electrooxidation and detection, and so the BDDGR electrode was used for the individual detection of CR and PQ. No lowering of the peak potential was noticed when modifying the BDD electrode.

**Figure 2:**
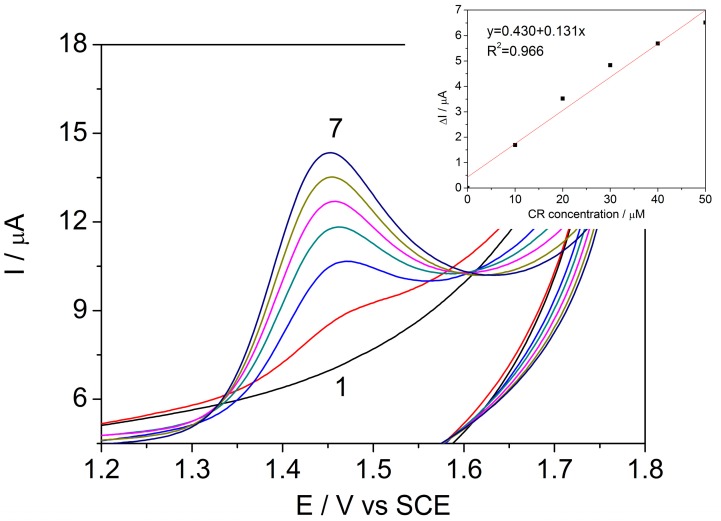
Anodic details of cyclic voltammograms recorded on the BDDGR electrode in acetic acid/sodium acetate buffer supporting electrolyte (curve 1) and in the presence of various CR concentrations: curves 2–7: 10–60 μM CR; potential scan rate: 0.05 V s^−1^; potential range: −1.0 to +1.75 V/SCE. Inset: Calibration plots of the currents recorded at E= +1.45 V vs. SCE versus CR concentrations.

Individual electrochemical detection of carbaryl on the BDDGR electrode was performed by cyclic voltammetry, in the potential range of −1.00 to +1.75 V/SCE. The well-defined oxidation peak described by the following reaction [24]:

CR^+^ → CR·^+^ + e^−^ (3)

was recorded at the potential +1.45 V/SCE (Figure 2) and increased linearly with CR concentration, allowing a good sensitivity for CR detection.

**Figure 3:**
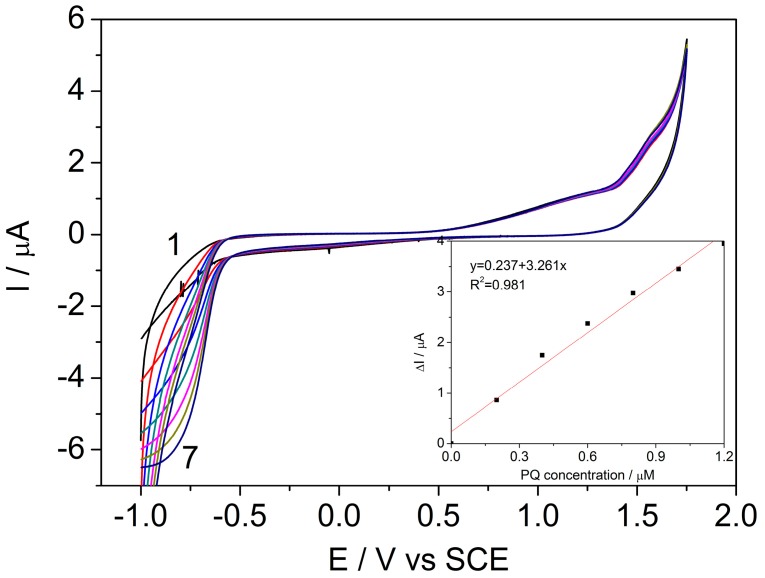
Cyclic voltammograms recorded on the BDDGR electrode in acetic acid/sodium acetate buffer supporting electrolyte (curve 1) and in the presence of various paraquat (PQ) concentrations: curves 2–7: 0.2–1.2 μM PQ; potential scan rate: 0.05 V s^−1^; potential range: −1.0 to +1.75 V/SCE. Inset: Calibration plots of the currents recorded at E = −0.78 V vs. SCE versus PQ concentrations.

In the same potential range, the individual electrochemical detection of PQ on the BDDGR electrode was performed using cyclic voltammetry (CV) in the acetate buffer. Electroreduction of PQ on the electrode surface was recorded at the potential −0.78 V/SCE, described by the reaction [29]:

PQ^2+^ + e^−^ → PQ^+^  (4)

and the modules of cathodic current increased also, linearly at increasing PQ concentration within the concentration range between 0.2 and 1.2 μM. The sensitivity of 46.12 μA μM^−1^ cm^−2^ and the limit of detection (LOD) of 10 nM were determined with the good correlation coefficient (R^2^) (Figure 3, Table 1).

#### 3.1.2. Differential Pulse Voltammetry

**Figure 4:**
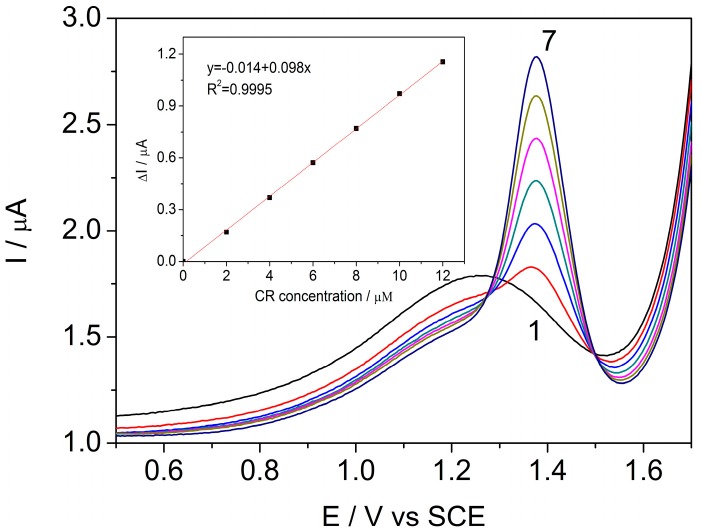
Differential pulse voltammograms recorded on the BDDGR electrode in acetic acid/sodium acetate buffer supporting electrolyte (curve 1) and in the presence of various CR concentrations: curves 2–7: 1–12 μM CR; step potential (sp) 5 mV, modulation amplitude (MA) 50 mV; potential range: −1.0 to +1.75 V/SCE. Inset: Calibration plots of the currents recorded at E = +1.42 V vs. SCE versus CR concentrations.

**Figure 5:**
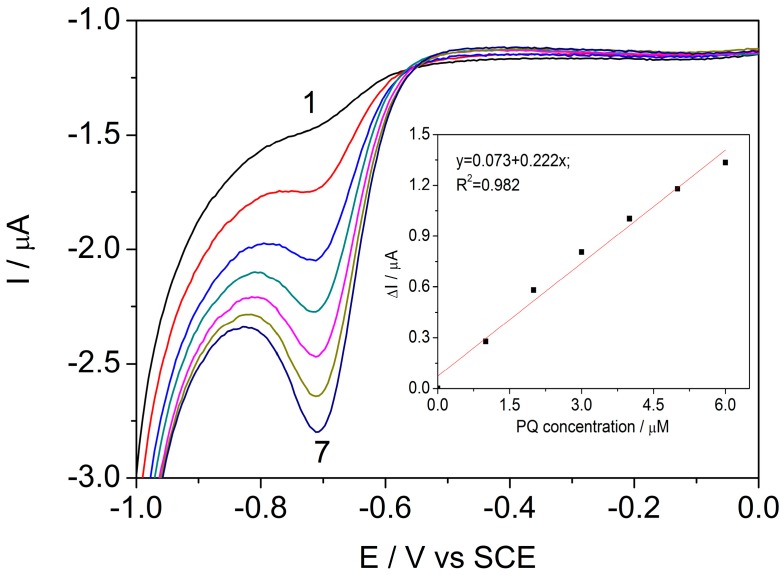
Differential pulse voltammograms recorded on the BDDGR electrode in acetic acid/sodium acetate buffer supporting electrolyte (curve 1) and in the presence of various PQ concentrations: curves 2–7: 1–6 μM PQ; step potential (sp) 5 mV, modulation amplitude (MA) 50 mV; potential range: −1.0 to +1.75 V/SCE. Inset: Calibration plots of the currents recorded at E = −0.71 V vs. SCE versus PQ concentrations.

It is well-known that differential pulse voltammetry (DPV) enhances the sensitivity of electroanalysis [30]. It is clear that the peak current is one of the most important aspects of electroanalysis due to the fact that it defines the sensitivity of the method. The peak current increase is related to the electrode kinetics, which depends on the operating conditions of step potential (sp), modulation time (mt) and modulation amplitude (MA) [31]. The DPV technique was applied at an sp of 5 mV, an mt of 0.004 s, and an MA of 50 mV, in the acetate buffer supporting electrolyte for the individual detection of CR (Figure 4) and PQ (Figure 5). The voltammograms recorded in these conditions revealed well-defined peaks for each analyzed pesticide.

However, even if the detection peak is more well-defined, the sensitivities recorded when the operating parameters were an sp of 5 mV and an MA of 50 mV were much lower in comparison with the above-reported operating conditions. This is explained by the compromise between the rate of transformation and depletion of the electroactive species, which are both promoted by fast electrode kinetics but with the opposite effect on the detection signal [31]. Consequently, further simultaneous detection analysis was performed using the DPV technique, with an sp of 6 mV and an MA of 800 mV. Using DPV testing, the best sensitivity of about 30 μA μM^−1^ cm^−2^ was reached for CR and PQ detection. (The results recorded by successive addition of CR and PQ are not shown here.) Under these operating conditions, the recorded sensitivity for the individual detection of CR was much higher than the one recorded by CV (Table 1). Another advantage of the DPV technique in CR detection was the shift in the detection potential towards the smaller overpotential value of +0.77 V/SCE. Electroanalytical parameters for individual detection of CR and PQ on the BDDGR electrode using CV and DPV techniques are collated in Table 1.

### 3.2. Simultaneous Detection of Carbaryl and Paraquat

#### 3.2.1. Simultaneous Detection of CR and PQ in Acetate Buffer

**Figure 6:**
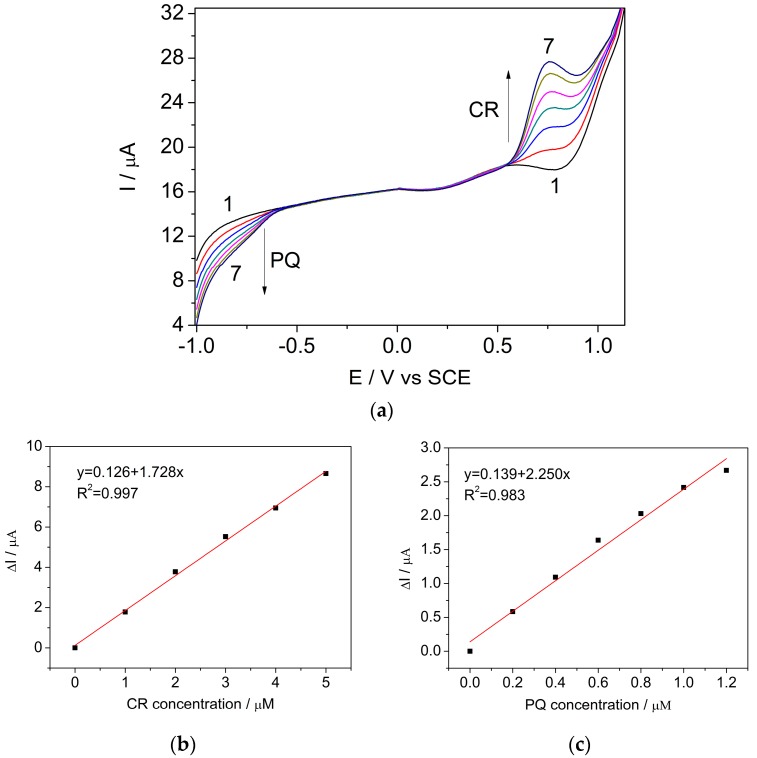
(**a**) Differential pulse voltammograms recorded on the BDDGR electrode in acetic acid/sodium acetate buffer supporting electrolyte (curve 1) and in the presence of various CR and PQ concentrations: curves 2–7: curve 2—1 μM CR + 0.2 μM PQ; curve 3—2 μM CR + 0.4 μM PQ; curve 4—3 μM CR + 0.6 μM PQ; curve 5—4 μM CR + 0.8 μM PQ; curve 6—5 μM CR + 1.0 μM PQ; curve 7—6 μM CR + 1.2 μM PQ; sp 6 mV, MA 800 mV; potential range: −1.0 to +1.75 V/SCE. Calibration plots of the currents recorded at (**b**) E = +0.74 V vs. SCE versus CR concentrations and (**c**) E = −0.80 V vs. SCE versus PQ concentrations.

Simultaneous detection of CR and PQ was further performed using the DPV technique (sp = 0.6 mV, MA = 800 mV) in the acetate buffer supporting electrolyte and in the presence of a mixture of CR and PQ at different concentrations, ranging from 1 to 6 μM CR and 0.2 to 1.2 μM PQ (Figure 6). The oxidation of carbaryl and reduction of paraquat on the BDDGR electrode surface allowed simultaneous detection of the target analytes, without any negative effect from their addition as a mixture (Figure 6), with the sensitivities presented in Figure 6b,c. The LOD of 0.07 μM for CR and 0.01 μM for PQ determined in the context of simultaneous detection also confirms that neither pesticide interferes with detection of the other. In addition, the good detection characteristics of this electrode are proved when compared with the results published for other electrodes, which have been tested only for the individual detection of each pesticide (see Table 2).

#### 3.2.2. Simultaneous Detection of CR and PQ in Natural Apple Juice

**Figure 7:**
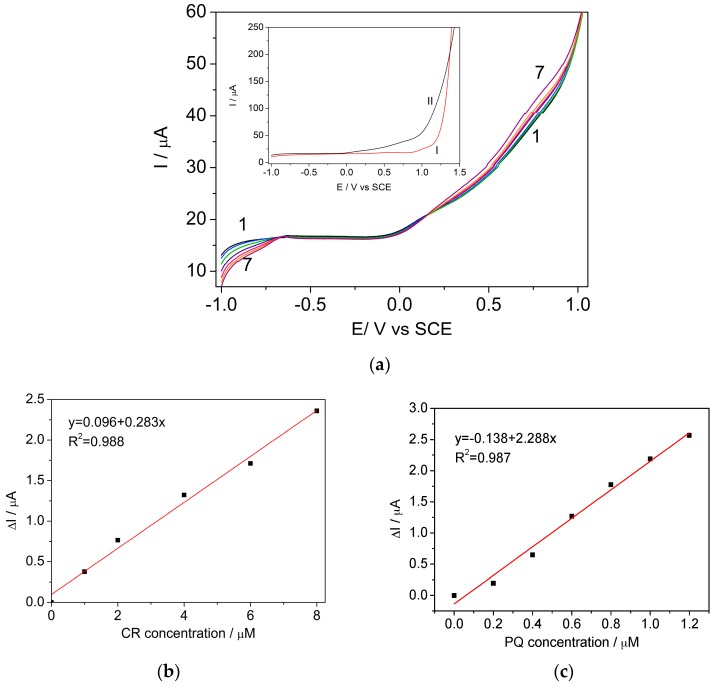
(**a**) Differential pulse voltammograms recorded on the BDDGR electrode in natural apple juice (curve 1) and in the presence of various CR and PQ concentrations: curves 2–7: curve 2—1 μM CR + 0.2 μM PQ; curve 3—2 μM CR + 0.4 μM PQ; curve 4—4 μM CR + 0.6 μM PQ; curve 5—6 μM CR + 0.8 μM PQ; curve 6—8 μM CR + 1.0 μM PQ; curve 7—10 μM CR + 1.2 μM PQ; sp 6 mV, MA 800 mV; potential range: −1.0 to +1.75 V/SCE. Inset: Differential pulse voltammograms recorded on the BDDGR electrode in acetic acid/sodium acetate buffer supporting electrolyte (curve I) and in natural apple juice (curve II). Calibration plots of the currents recorded at (**b**) E = +0.77 V vs. SCE versus CR concentrations and (**c**) E = −0.83 V vs. SCE versus PQ concentrations.

Carbaryl and paraquat are widely used in apple fruit culture. The proposed method was applied to detect CR and PQ in natural apple juice under the optimized conditions above-described. Filtered fresh apple juice was spiked with certain concentrations of CR and PQ and added directly into the electrochemical cell. The DPV technique was operated at an sp of 6 mV and an MA of 800 mV for the simultaneous detection of carbaryl and paraquat mixture on the BDDGR electrode. Simultaneous detection in the apple juice, without any supporting electrolyte added, was achieved. The sensitivity for PQ detection is similar to the one recorded in acetate buffer: 32.36 μA μM^−1^ cm^−2^ in juice vs. 31.86 μA μM^−1^ cm^−2^ recorded in acetate buffer (Table 3). In the anodic part, the oxidation of CR in natural juice takes place at the same potential as the one recorded in acetate buffer, but the recorded electroanalytical parameters in terms of sensitivity and limit of detection are inferior to the one recorded in acetate buffer. This phenomenon is likely due to an intense background current corresponding to the natural apple juice in the anodic part of the voltammogram (Inset of Figure 7a), which may be related to other matrix electroactive components: in the case of apples, those components will be ascorbic acid (vitamin C) and sucrose [35]. Linear dependences between recorded current and concentration of the carbaryl and paraquat in juice were obtained (Figure 7b,c).

These results impose a calibration on the natural apple juice before the application of BDDGR-based DPV. The reproducibility of the proposed methodology was assessed based on five different measurements [36] in the same fresh apple juice containing 8 × 10^−6^ mol L^−1^ CR and 1 × 10^−6^ mol L^−1^ PQ, and the coefficients of variation of 5.00 and 1.50% respectively were achieved. This confirms the reproducibility of the proposed detection method. The proposed detection method was applied to 10 different solutions characterized by the same above-presented composition [25] and the coefficients of variation of 2.50% for CR and 1.20% for PQ confirm the repeatability of the method.

## 4. Conclusions

A BDD electrode modified with graphene, named BDDGR in this study, exhibited superior electroanalytical performance for the oxidation of carbaryl and reduction of paraquat at its surface in comparison to a simple BDD electrode, due to the electrocatalytic effect of graphene. This fact allowed the achievement of very good results for individual and simultaneous detection by cyclic and differential-pulse voltammetric techniques. The DPV technique, operated with an sp of 6 mV and an MA of 800 mV, allowed the individual and simultaneous detection of carbaryl and paraquat with a sensitivity of about 30 μA μM^−1^cm^−2^, and the lowest limit of detection of 10 nM for paraquat and 70 nM for carbaryl. The same analytical procedure was applied to simultaneously detect carbaryl and paraquat in natural fresh apple juice, where it was found that Vitamin C and sucrose presence exerted interference on the sensitivity of carbaryl detection. No interference on paraquat detection was found from the spiked apple fruit sample analyzed without any treatment. A prior calibration of the BDDGR electrode in fresh juice demonstrated the reproducibility and the repeatability of this proposed method for the simultaneous detection of carbaryl and paraquat in apple juice. Thus, the use of BDDGR in conjunction with the DPV technique to simultaneously detect carbaryl and paraquat seems to present great potential due to its application in a variety of different natural matrices.

## Figures and Tables

**Table 1:** Electroanalytical parameters recorded on the BDDGR electrode for individual detection of CR and PQ.

Technique	Target Analyte	Parameters	Potential/V	Sensitivity (μA μM^−1^ cm^−2^)	*LOD* (μM)	*LOQ* (μM)	R^2^
CV	CR	v = 0.05 Vs^−1^	+1.47	1.85	0.14	0.46	0.966
	PQ	v = 0.05 Vs^−1^	−0.78	46.12	0.01	0.04	0.981
DPV	CR	sp = 6 mV, MA = 800 mV	+0.74	30.5	0.07	0.23	0.985
		sp = 5 mV, MA = 50 mV	+1.42	1.39	0.16	0.55	0.999
	PQ	sp = 6 mV, MA = 800 mV	−0.80	30.8	0.04	0.13	0.993
		sp = 5 mV, MA = 50 mV	−0.71	3.14	0.02	0.08	0.982

**Table 2:** Comparison of electroanalytical parameters for CR and PQ detection obtained at the BDDGR electrode with other published electrode material.

Electrode	Analyte		Linear Range (μM)	*LOD* (μM)	Reference
	CR	PQ			
MWCNTs/Cobalt Phtalocyanine/GCE	x	-	0.33–6.61	0.005	[24]
Graphene oxide-ionic liquid/GCE	x	-	0.10–12	0.02	[32]
BDD	x	-	1–30	0.041	[28]
AuNPs/DNA/GE	-	x	0–100	1.30	[33]
MWCNTs-DHP/GCE	-	x	0.20–1.70	0.026	[34]
PPY-NGE/GCE	-	x	0.05–2	0.041	[29]
BDDGR	x	x	1–6; 0.2–1.2	0.07; 0.01	This work

**Table 3:** Electroanalytical parameters recorded on the BDDGR electrode for simultaneous detection of CR and PQ by the DPV technique.

Supporting Electrolyte	Target Analyte	Potential(V)	Sensitivity (μA μM^−1^ cm^−2^)	*LOD* (μM)	*LOQ* (μM)	R^2^
**Acetate Buffer**	CR	+0.74	33.27	0.07	0.23	0.997
	PQ	−0.80	31.83	0.01	0.02	0.983
**Natural Apple Juice**	CR	+0.77	4.00	0.17	0.59	0.988
	PQ	−0.83	32.36	0.04	0.14	0.987
